# Supplementary material for: Layer-specific morphological and molecular differences in neocortical astrocytes and their dependence on neuronal layers
Source: Nat Commun. 2018 Apr 24;9:1623. doi: 10.1038/s41467-018-03940-3 (PMC5915416; doi:10.1038/s41467-018-03940-3)
Supplement: Supplementary file 1 — Supplementary Information [file 41467_2018_3940_MOESM1_ESM.pdf]

Supplementary Information

Layer-specific morphological and molecular differences in neocortical astrocytes and their dependence on neuronal layers

Lanjakornsiripan et al.

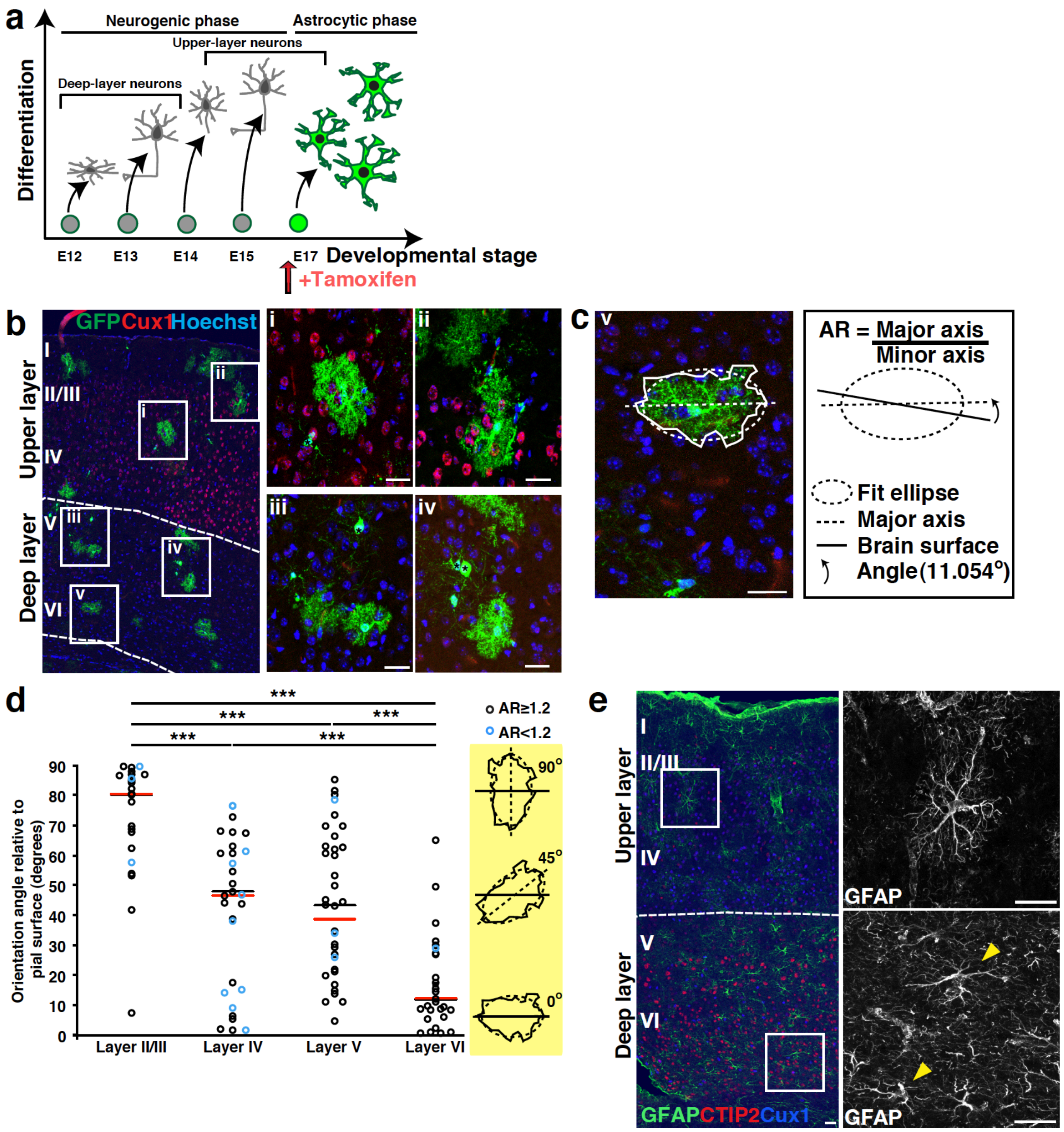

Supplementary Figure 1. Differences of cell orientation in neocortical astrocytes in the somatosensory area.

(**a**) Schematic representation of astrocyte labeling in Sox9-CreER^{T2};CAG-CAT-eGFP mice. Astrocytes were sparsely labeled with GFP after administration of a low dose of tamoxifen to dams at the gliogenic stage (embryonic day 17). (**b**) Immunostaining for GFP and Cux1 in coronal sections of the Sox9-CreER^{T2};CAG-CAT-eGFP mouse brain at P120. Nuclei were stained with Hoechst 33342. The boxes in the left panel are shown at higher magnification on the right. Asterisks mark oligodendrocytes. Scale bars, 25 μm. (**c**) Schematic representation of cell orientation measurement.

An outline of a cell was drawn and an ellipse fitted with the use of Fiji software (shown by the white polygon and dashed ellipse, respectively). The aspect ratio (AR) of the fitted ellipse and its angle of orientation relative to the brain surface were also calculated with Fiji software. Scale bar, 25 μm. (**d**) Quantification of the angle of orientation for astrocytes in each layer at P120. Data are shown for 118 cells from two brains, with the red and black bars indicating the median values of each data set and those for cells with an AR of ≥1.2, respectively. Black open circles (AR of ≥1.2) and blue open circles (AR of <1.2) indicate elliptic and roundlike astrocytes, respectively. ***$P < 0.001$ (one-way ANOVA followed by Bonferroni's test). (**e**) Immunofluorescence staining for GFAP, CTIP2, and Cux1 on coronal sections of the wild-type mouse brain at P60. GFAP staining in the boxed regions of the left panel is shown at higher magnification on the right, revealing a vertically oriented, highly branched upper-layer astrocyte (top right) and two horizontally oriented deep-layer astrocytes (arrowheads) with few processes (bottom right). The dashed line indicates the boundary between upper and deep layers. Scale bars, 25 μm.

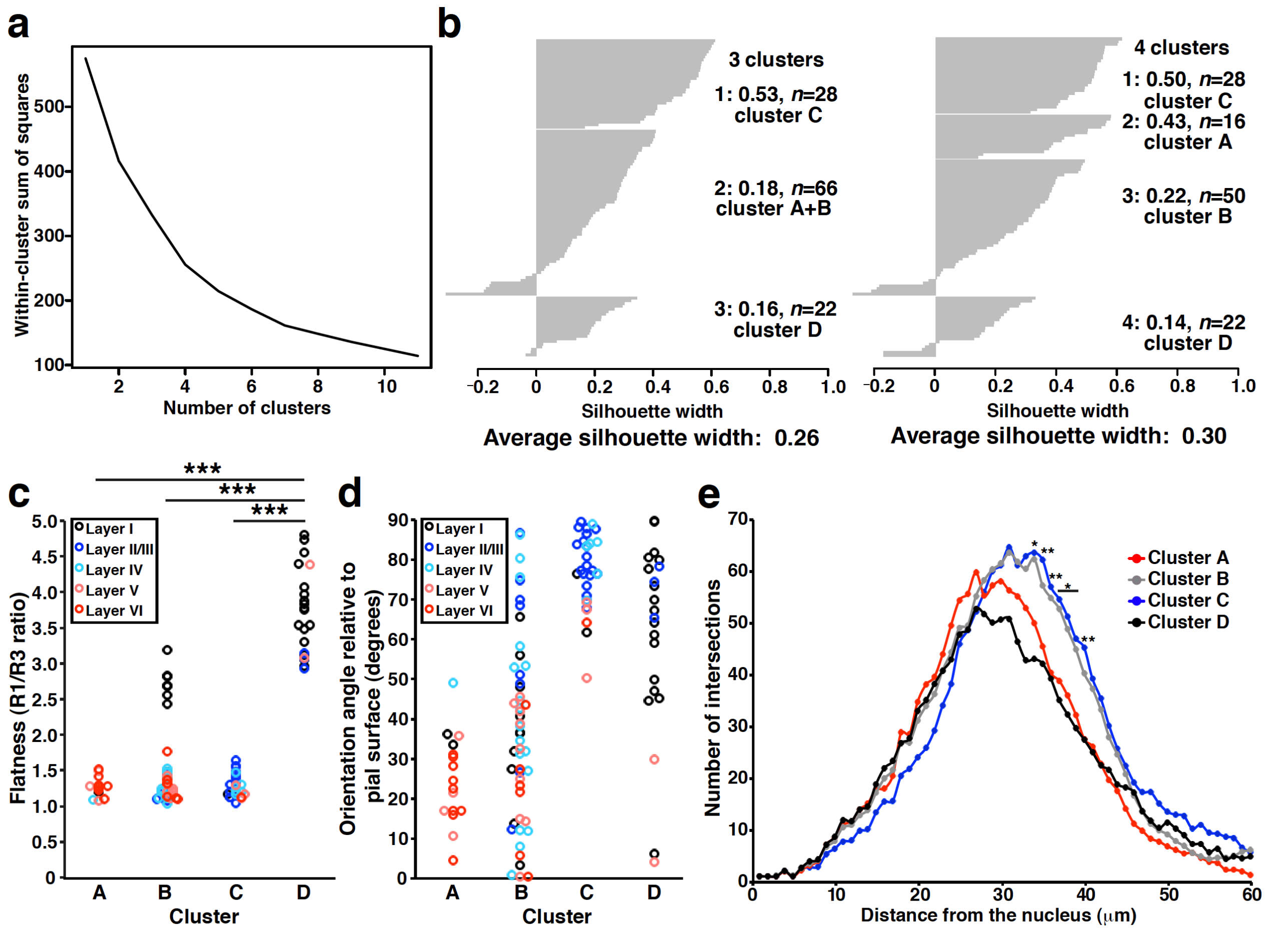

Supplementary Figure 2. Optimal classification of neocortical astrocytes into four clusters. (**a**) Total within-cluster sum of squares (intracluster distance) showing the maximum decrease when the number of clusters is 3 or 4. (**b**) Silhouette analysis for evaluation of the similarity within clusters when neocortical astrocytes are classified into three clusters (left) or four clusters (right). Silhouette width was used to identify natural groups from artificially imposed groups, with this parameter being higher when data points are more similar within a group. (**c**–**e**) Representative morphological features including flatness (**c**), angle of orientation relative to the brain surface (**d**), and extent of process arborization as evaluated by 3D Sholl analysis (**e**) for astrocytes in clusters A through D. *$P < 0.05$, **$P < 0.01$, ***$P < 0.001$ (one-way ANOVA followed by Bonferroni's test (**c**) or two-way ANOVA followed by Bonferroni's test (**e**)) for the indicated comparisons (**c**) or for cluster A versus cluster C (**e**).

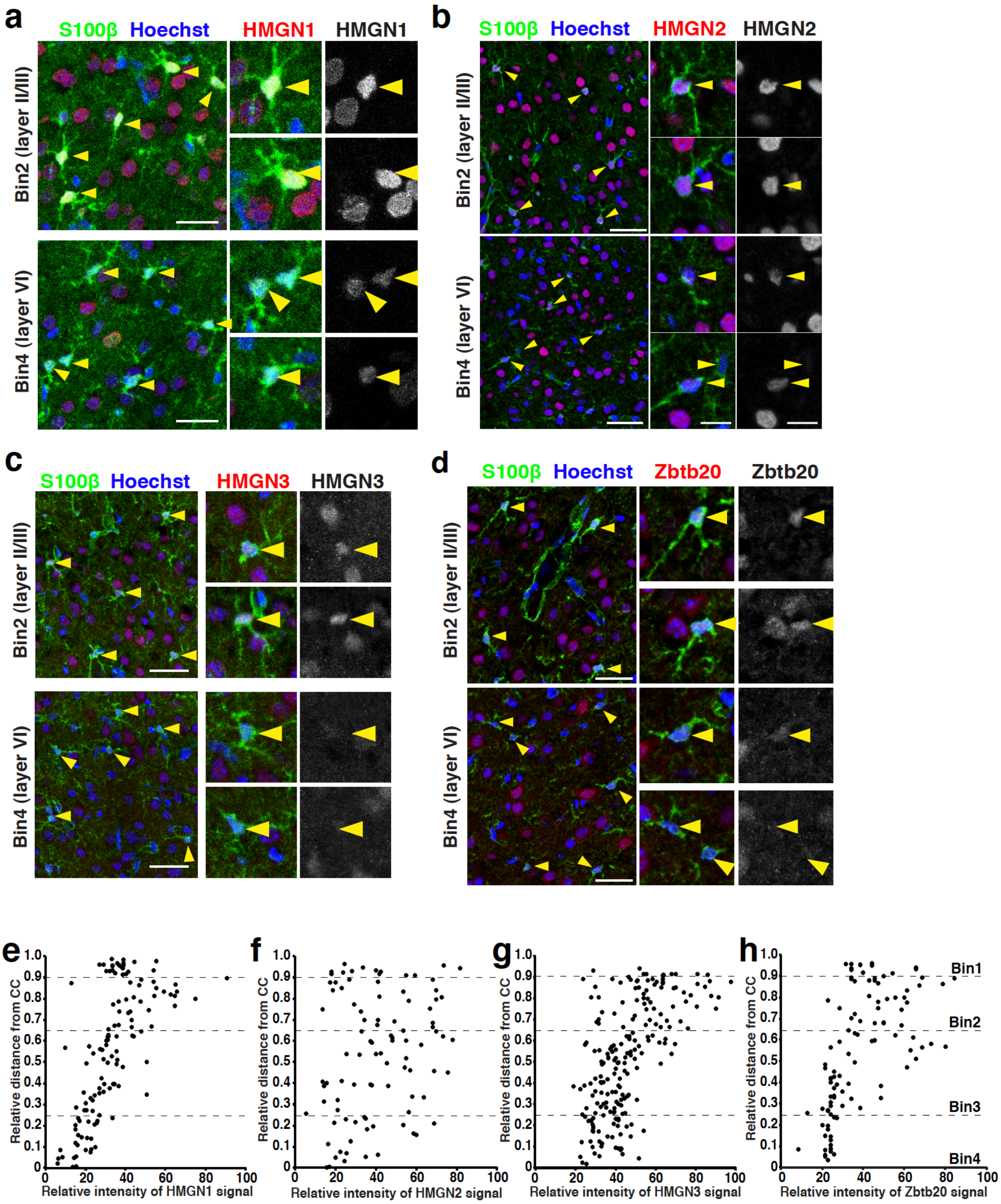

Supplementary Figure 3. Layer-specific expression of HMGN1–3 and Zbtb20 in neocortical astrocytes.

(**a-d**) Coronal sections of the Aldh1l1-eGFP mouse brain at P16 were immunostained for the astrocyte marker S100β and either HMGN1 (**a**), HMGN2 (**b**), HMGN3 (**c**), or Zbtb20 (**d**). Nuclei were stained with Hoechst 33342. Arrowheads indicate S100β-positive astrocytes. Scale bars, 25 μm.

(**e-h**) Representative data of HMGN1 (**e**), HMGN2 (**f**), HMGN3 (**g**), and Zbtb20 (**h**) signal intensities in the nucleus of individual astrocytes in a coronal section. Astrocyte position in the cortical layers is expressed as relative distance from the corpus callosum (CC) to the pia, with dashed lines indicating the boundaries between bins: bin1, 0.9–1.0; bin2, 0.65–0.9; bin3, 0.25–0.65; and bin4, 0–0.25.

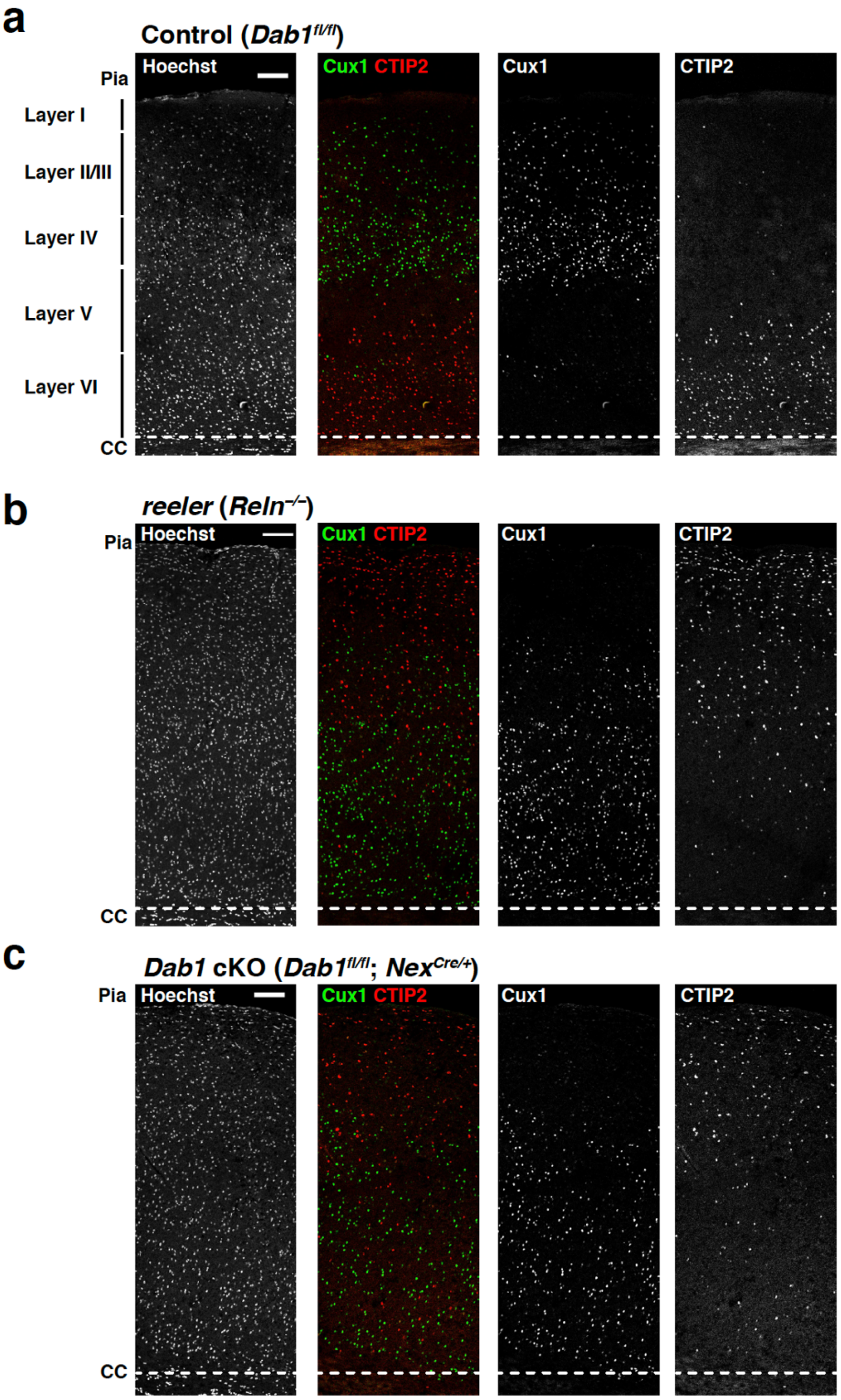

Supplementary Figure 4. Disruption of neuronal laminar structure in *reeler* and *Dab1* cKO mice.

Coronal sections of the somatosensory cortex (S1) of control (*Dab1*$^{\mathrm{fl/fl}}$) (**a**), *reeler* (**b**), and *Dab1* cKO (*Dab1*$^{\mathrm{fl/fl}}$;*Nex*$^{\mathrm{Cre/+}}$) (**c**) mice at P60 to P70 were subjected to immunofluorescence staining for Cux1 and CTIP2 as neuronal layer markers. Nuclei were stained with Hoechst 33342. CC, corpus callosum. Scale bars, 100 μm.

Supplementary Table 1. Multimodality index (MMI) of 24 morphometric parameters. Asterisks indicate parameters used as criteria in cluster analysis.

**Parameter**	**Multimodality index**
Volume	0.363
Surface area	0.433
Compactness	0.459
Sphericity	0.385
R1	0.442
R2	0.363
R3	0.342
Elongation*	0.591
Flatness*	0.812
Spareness	0.417
XY*	0.559
Relative XZ*	0.562
Relative YZ*	0.667
Convex hull volume	0.367
Convex hull surface area	0.352
Solidity	0.404
Convexity	0.302
Microtubule volume	0.413
Microtubule surface area	0.403
Ending Sholl radius	0.452
Sum of intersections	0.389
Max intersections	0.433
Max intersecting radius	0.345
Ramification index	0.431
