# Supplementary material for: Layer-specific morphological and molecular differences in neocortical astrocytes and their dependence on neuronal layers
Source: Nat Commun. 2018 Apr 24;9:1623. doi: 10.1038/s41467-018-03940-3 (PMC5915416; doi:10.1038/s41467-018-03940-3)
Supplement: Supplementary file 3 — Description of Additional Supplementary Files [file 41467_2018_3940_MOESM3_ESM.pdf]

Description of Additional Supplementary Files

File Name: Supplementary Data 1
Description: RNA-seq analysis, upper-layer enriched gene list.

File Name: Supplementary Data 2
Description: RNA-seq analysis, deep-layer enriched gene list.

File Name: Supplementary Data 3
Description: RNA-seq analysis, astrocyte-enriched gene list.
